# Intratumoral acidosis fosters cancer-induced bone pain through the activation of the mesenchymal tumor-associated stroma in bone metastasis from breast carcinoma

**DOI:** 10.18632/oncotarget.17091

**Published:** 2017-04-13

**Authors:** Gemma Di Pompo, Silvia Lemma, Lorenzo Canti, Nadia Rucci, Marco Ponzetti, Costantino Errani, Davide Maria Donati, Shonagh Russell, Robert Gillies, Tokuhiro Chano, Nicola Baldini, Sofia Avnet

**Affiliations:** ^1^ Orthopaedic Pathophysiology and Regenerative Medicine Unit, Istituto Ortopedico Rizzoli, Bologna, Italy; ^2^ Department of Biomedical and Neuromotor Sciences, University of Bologna, Bologna, Italy; ^3^ Department of Biotechnological and Applied Clinical Sciences, University of L’Aquila, L’Aquila, Italy; ^4^ Orthopaedic Oncology Surgical Unit, Istituto Ortopedico Rizzoli, Bologna, Italy; ^5^ Department of Imaging Research, H. Lee Moffitt Cancer Center and Research Institute, Tampa, FL, USA; ^6^ Department of Clinical Laboratory Medicine, Shiga University of Medical Science, Otsu, Shiga, Japan

**Keywords:** cancer-induced bone pain, intratumoral acidosis, tumor-associated stroma, hyperalgesia

## Abstract

Cancer-induced bone pain (CIBP) is common in patients with bone metastases (BM), significantly impairing quality of life. The current treatments for CIBP are limited since they are often ineffective. Local acidosis derived from glycolytic carcinoma and tumor-induced osteolysis is only barely explored cause of pain. We found that breast carcinoma cells that prefer bone as a metastatic site have very high extracellular proton efflux and expression of pumps/ion transporters associated with acid-base balance (MCT4, CA9, and V-ATPase). Further, the impairment of intratumoral acidification via V-ATPase targeting in xenografts with BM significantly reduced CIBP, as measured by incapacitance test. We hypothesize that in addition to the direct acid-induced stimulation of nociceptors in the bone, a novel mechanism mediated by the acid-induced and tumor-associated mesenchymal stroma might ultimately lead to nociceptor sensitization and hyperalgesia. Consistent with this, short-term exposure of cancer-associated fibroblasts, mesenchymal stem cells, and osteoblasts to pH 6.8 promotes the expression of inflammatory and nociceptive mediators (NGF, BDNF, IL6, IL8, IL1b and CCL5). This is also consistent with a significant correlation between breakthrough pain, measured by pain questionnaire, and combined high serum levels of BDNF and IL6 in patients with BM, and also by immunofluorescence staining showing IL8 expression that was more in mesenchymal stromal cells rather than in tumors cells, and close to LAMP-2 positive acidifying carcinoma cells in BM tissue sections.

In summary, intratumoral acidification in BM might promote CIBP also by activating the tumor-associated stroma, offering a new target for palliative treatments in advanced cancer.

## INTRODUCTION

Skeletal involvement is a frequent and troublesome complication affecting many patients with advanced cancer. Bone is the third most common metastatic site [1]: up to 85% of patients that die from breast, prostate, or lung cancer have bone involvement at autopsy [2, 3]. Once tumor cells become housed in the skeleton, the disease is usually considered incurable, and treatment is only palliative. One of the most intolerable symptoms is cancer-induced bone pain (CIBP), which has a strong impact on quality of life. CIBP causes difficulty in ambulation, neurologic deficits, depression, and anxiety. Current therapies based on radiotherapy, non-steroidal anti-inflammatory drugs (NSAIDs), bisphosphonates, and opioids are often in-effective and cause unacceptable side effects [3–5]. Opioids often cause excessive sedation and lack of responsiveness, and a strong evidence base for the use of NSAIDs is still lacking [6]. Therefore, new and effective palliative measures are required to maintain an acceptable quality of life.

A novel approach focusing on recent insights into the molecular mechanisms of CIBP and the peculiar features of the microenvironment of bone metastases (BM) might help improve therapy options. The pathophysiology of bone pain is unique and complex [7]. CIBP is a mixed type of chronic pain involving both inflammatory nociceptive and neuropathic pain, and it can be breakthrough, a transitory flare of pain in the setting of chronic pain managed with opioid drugs. Neuropathic pain is evoked by the compression and invasion of sensory nerves by cancer cells, whereas mechanical pain is induced by the stretching of the periosteum for tumor expansion, or by microfractures that occur as a consequence of increased bone resorption [2, 8]. Sensory neurons (nociceptors) densely innervate the bone marrow and the periosteal surfaces of the cortical bone and can sense diverse noxious stimuli. Nociceptors are activated through acid-sensing receptors, [i.e, acid-sensing ion channels (ASICs) and the transient receptor potential channel-vanilloid subfamily members (TRPVs)] [9]. Therefore, protons are important mediators of nociceptor stimulation to evoke an allogenic signal [10, 11]. Local acidosis is common in BM and is independent of the inflammatory reaction, because it derives from the extracellular acidification caused by *inter alia*, mesenchymal stromal cells, bone-resorbing osteoclasts, and metastatic cancer cells. The relative contributions of these diverse sources of acid to CIBP are not well characterized. Because of their elevated level of aerobic glycolysis, aggressive cancer cells secrete substantial amounts of protons [12] through an armamentarium of pumps and transporters, including vacuolar H+-ATPase (V-ATPase) that is directly associated with tumor invasion and metastasis [12, 13]. Further, tumor can induce osteoclasts that dissolve bone through the secretion of large amounts of protons, also via V-ATPase, into the resorption lacunae, reaching pH levels around 4.5 [14]. Thus, these large amounts of protons in the extracellular space can directly stimulate nociceptors through acid-sensing ion channels. We explored intratumoral acidification as a novel target for palliative treatment in patients with BM also by investigating on the role of mesenchymal stromal cells in mediating nociceptive stimulation in the acidic BM microenvironment.

## RESULTS

### Breast carcinoma cells that prefer the bone as a metastatic site strongly acidify the extracellular microenvironment

**Figure 1:**
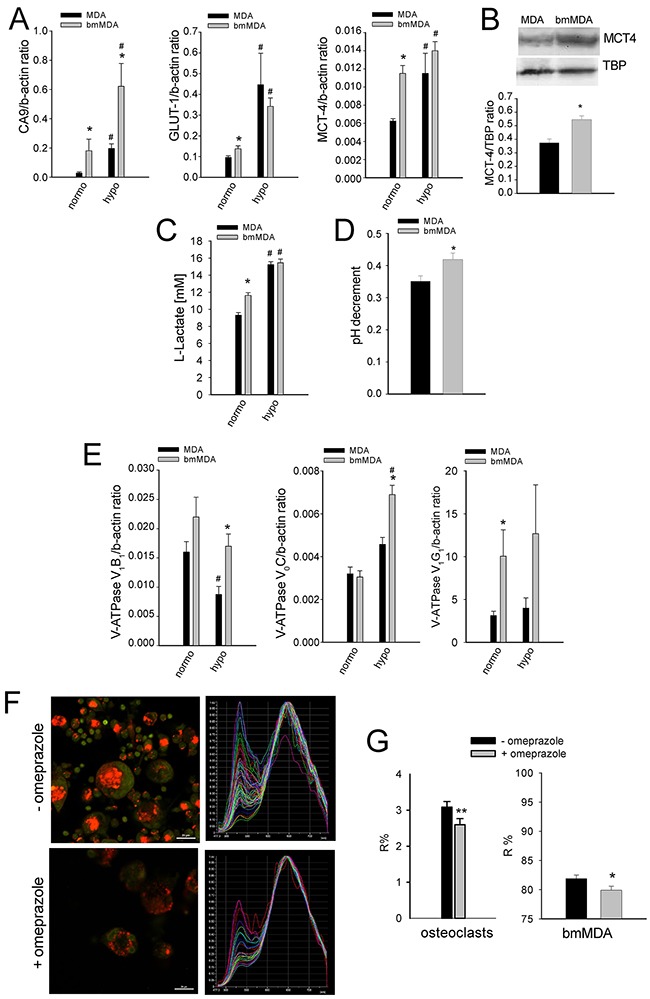
Breast carcinoma bmMDA cells show a higher glycolytic metabolism with a higher extracellular acidification activity than the parental MDA **(A)** mRNA analysis of CA9, GLUT1, and MCT4 expression under different O_2_ tensions. (N = 2 technical and N = 3 biological replicates for GLUT1 and MCT4, and N = 2 technical and N = 2 biological replicates for CA9). **(B)** Western blotting analysis of MCT4 (upper panel, representative experiment; lower panel, densitometric quantification of N = 4 biological replicates); **(C)** L-Lactate concentration in the culture supernatants under different O_2_ tensions (N = 4 technical replicates); **(D)** pH decrement of the culture supernatant by electrode measurement (N = 6 biological replicates); **(E)** mRNA analysis of the expression of V-ATPase subunits under different O_2_ tensions (normoxia, normo; hypoxia, hypo) (N = 2 technical and N = 3 biological replicates for V_1_B_2_ and V_1_G_1_, and, N = 2 technical and N = 2 biological replicates for V_0_C); **(F)** Representative image of acridine orange staining (left panel) and graphical representation of the emission spectrum by confocal analysis (right panel) of all the lysosomes per cell of one representative osteoclast in the treated or not treated cultures with omeprazole; **(G)** lysosomal pH of cells treated with omeprazole as evaluated by acridine orange staining and spectral confocal microscopy and expressed as R% (N = 14 biological replicates). Mean ± SE, **p < 0.01 and *p < 0.05 for bmMDA vs MDA, and ^#^ *p* < 0.05 for normoxia (normo) vs hypoxia (hypo).

Breast carcinoma cells strongly acidify the tumor microenvironment [15]. Such acidification might promote the pathogenic process in bone; however, the association between the ability to localize in the bone and the ability to secrete high levels of protons has never been evaluated. We compared the MDA cell line with the bmMDA clone, which is more prone to form BM when injected into the left ventricle of nude mice [16]. Compared with the parental MDA cells, the bmMDA cells showed higher mRNA (Figure 1A) expression of key regulators of intracellular pH [12, 17], such as carbonic anhydrase 9 (CA9; mRNA *p* = 0.0209) and monocarboxylate transporter 4 (MCT4; mRNA *p*=0.0209, protein *p*=0.0495), and glucose transporter 1 (GLUT-1; mRNA *p*=0.045), an indirect marker of glycolysis. Results were confirmed also at protein level for MCT4 (Figure 1B). Moreover, the supernatants of the bmMDA cultures displayed greater release of L-Lactate (*p*=0.0209; Figure 1C). Further, a reduced-oxygen condition enhanced the mRNA expression of the analyzed genes and the release of L-Lactate (Figure 1A and 1C).

In association with the increased glycolytic activity and lactate release, the bmMDA cells accumulated more protons in the lysosomes and extruded more protons into the extracellular space than the parental MDA cells. Compared with the MDA cells, the bmMDA cells has significantly more pumped protons (pH decrement, *p*=0.0476 Figure 1D), and expressed a higher level of the V-ATPase, especially under hypoxia for the V_1_B_2_ and V_0_C subunits, or already at normoxia for the V_1_G_1_ subunit (*p*=0.0209, *p*=0.0209, and *p*=0.0495, respectively; Figure 1E). These results suggested that the breast carcinoma cells that are more prone to metastasize to the bone are positively selected from the original tumor-cell population based on their pronounced aptitude to release protons outside the cell. The extracellular acidification by breast cancer cells and tumor-induced osteoclasts could thus be directly responsible for the stimulation of acid-sensing ion channels in the nociceptors that innervate the bone, ultimately leading to bone pain.

### The targeting of intratumoral acidification reduces CIBP

**Figure 2:**
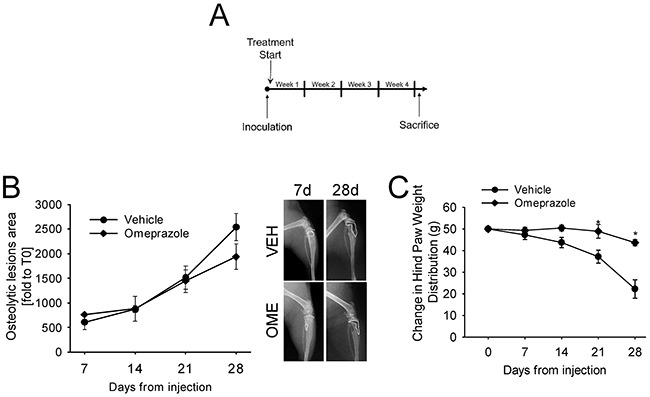
*In vivo* treatment with omeprazole bmMDA cells were inoculated by intratibial injection and then mice were treated with omeprazole (OME) or vehicle (VEH, 0.9% NaCl). **(A)** Schematic representation of the treatment; **(B)** Analysis of osteolytic areas evaluated via software image analyses (left panel) and representative x-ray scans (right panels, dashed line: osteolytic area); **(C)** Pain was evaluated by measuring changes in hind paw weight distribution (weight bearing) by using an incapacitance tester at different time points after tumor injection and during the treatment with omeprazole. Mice were placed in the measurement chamber of the incapacitance tester in an upright position, with the non-inoculated and inoculated limbs placed on 2 separate micro-scales. The changes in hind paw weight distribution was calculated by determining the difference (expressed as percent difference) in the amount of weight **(G)** between the left and right limbs. Mean ± SE, *p<0.05 vs vehicle treated. N=6 per group.

Omeprazole, a gastric H+/K(+)-ATPase inhibitor that can also block V-ATPase [18] has recently sparked great interest in relation to extracellular acidification in tumors. Acridine orange staining and spectroconfocal microscopy verified that the treatment effectively inhibited lysosomal acidification both in human osteoclasts and in bmMDA cells (*p*=0.023 and *p*=0.0373, respectively; Figure 1F and 1G, Supplementary Figure 1). We evaluated the effect of omeprazole on CIBP *in vivo* (Figure 2) with intratibially injected bmMDA. The omeprazole treatment did not alter tumor-promoted osteolysis (Figure 2B) since, with the exception of the last endpoint, a non-significant trend of reduction in the area of osteolytic lesions was observed (Figure 2B). However, omeprazole treatment significantly reduced CIBP, as detected by the incapacitance tester. This type of assay measures changes in hind paw weight distribution between the right (tumor bearing) and left (contralateral control) limbs, a parameter that is correlated with pain (p=0.023; Figure 2C) [53].

### Role of mesenchymal cells in acid-induced CIBP and hyperalgesia

**Figure 3:**
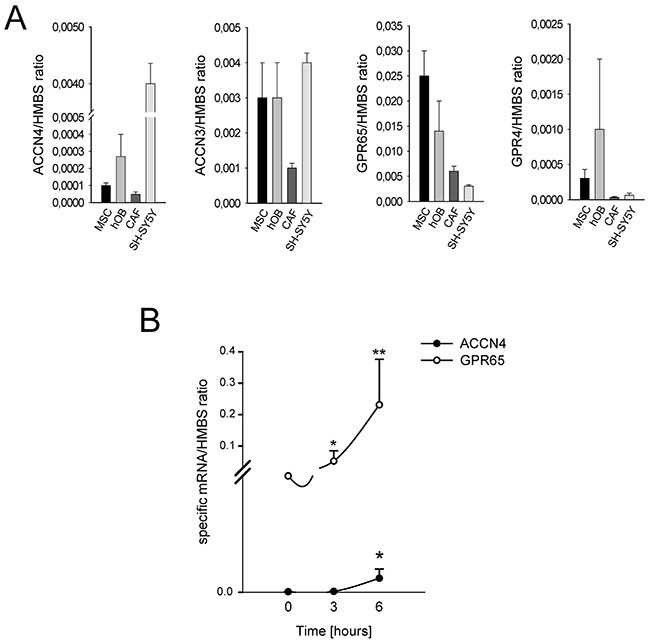
Expression of ion channels in BM-associated mesenchymal cells **(A)** mRNA analysis of the basal level of expression of ACCN4, ACCN3, GPR65, and GPR4 in MSCs, HOBs, and CAFs. Mean ± SE (N = 2 technical replicates and N = 3 biological replicates for MSCs and HOBs, and N = 2 technical replicates and N = 2 biological replicates for CAFs); **(B)** ACCN4 and GPR65 mRNA analysis of MSCs after 6 hours of culture in acidic medium. Mean ± SE (N = 2 technical replicates and N = 3 biological replicates); **p < 0.01, and *p < 0.05.

We investigated the mechanism of the treatment-resistant and hyperalgesic component of CIBP. We hypothesized that the tumor-associated mesenchymal stroma in the BM is involved in pain-related biological processes. As *in vitro* models, we used HOBs and MSCs from healthy donors, and CAFs isolated from BM biopsies. First, we assessed whether the mesenchymal cells of the BM microenvironment can detect the lowering of extracellular pH. MSCs, HOBs, and CAFs express ASIC4/ACCN4, ASIC3/ACCN3, GPR65, and GPR4 at levels often comparable to or even higher to those expressed by cells of neuronal origin (Figure 3A). Notably, the ASIC4/ACCN4 and GPR65 levels in MSCs were further increased after a short (6 h) incubation under acidosis (*p*=0.0064 and *p*=0.025, respectively; Figure 3B). Thus, these data indicate that BM-associated mesenchymal cells have mechanisms in place to perceive the acidification of the BM microenvironment.

**Figure 4:**
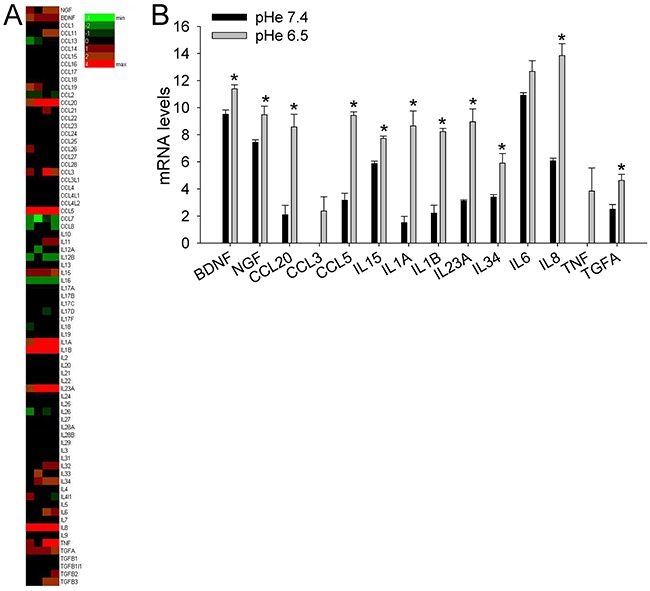
Short exposure to acidosis induces the release of inflammatory and nociceptive mediators in mesenchymal stromal cells of the BM microenvironment (deep sequencing of MSCs) **(A)** Heat map representation of the fold increase, by deep-sequencing analysis, of the mRNA levels of inflammatory and nociceptive mediators of normal stromal cells (4 lots of BM-MSCs) after short-term acidosis (pH 6.5) compared to physiological medium (pH 7.4). Colors on the heat map indicate the log2 ratios of expression (representing normalized read counts). Red, upregulation; green, downregulation; **(B)** Short-term acidosis activates inflammatory and nociceptive mediators in normal mesenchymal cells. Graphical representation of transcriptional fold increases after short-term acidosis (24 hrs at pH 6.5) of inflammatory and nociceptive mediators genes, in respect to physiological medium (pH 7.4), in normal stromal cells as revealed by deep-sequencing analysis. Mean ± SE (N = 4 lots of BM-MSCs). *p < 0.05.

**Figure 5:**
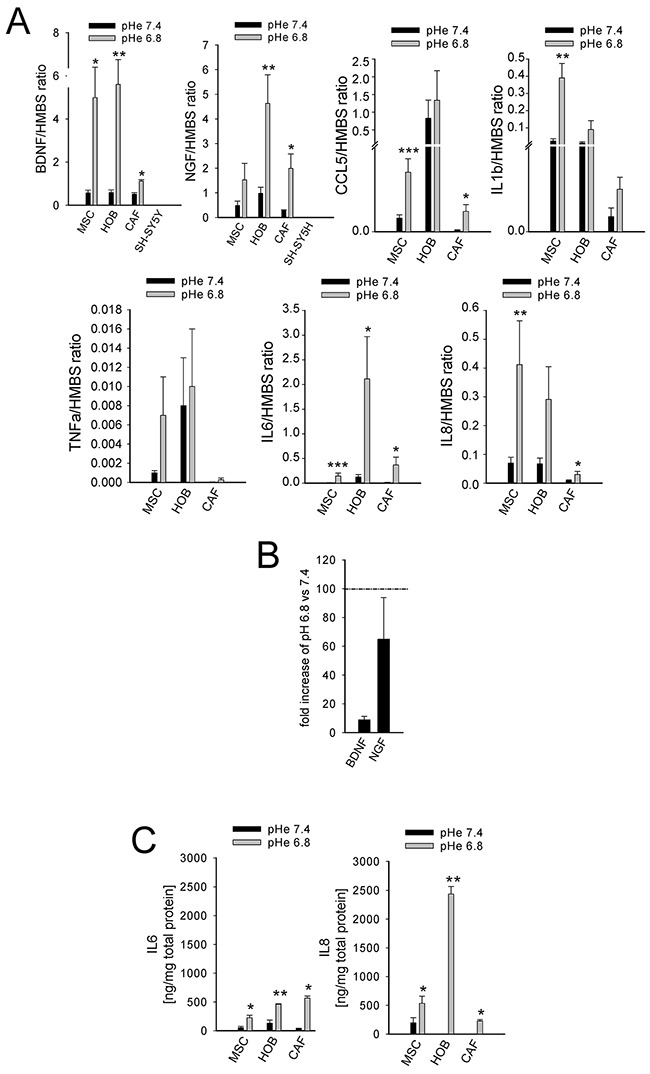
Short exposure to acidosis induces the release of inflammatory and nociceptive mediators in mesenchymal stromal cells of the BM microenvironment (MSCs, HOBs, and CAFs) **(A)** mRNA analysis of inflammatory and nociceptive mediators. Mean ± SE (N = 3 lots with N = 2 technical and N = 2 biological replicates each lot for MSCs and HOBs, and only 1 lot for CAFs with the same number of biological and technical replicates) ***p<0.001, **p < 0.01, and *p < 0.05; **(B)** mRNA expression of BDNF and NGF in bmMDA cells in respect to HOBs exposed for 24 hrs to low pH (= K, 100%); **(C)** ELISA quantification of IL6 and IL8 in the culture supernatants. Mean ± SE (N = 3 lots with N = 2 technical each lot for MSCs and HOBs, and only 1 lot for CAFs with N = 2 technical and N = 2 biological replicates) **p < 0.01 and *p < 0.05.

**Figure 6:**
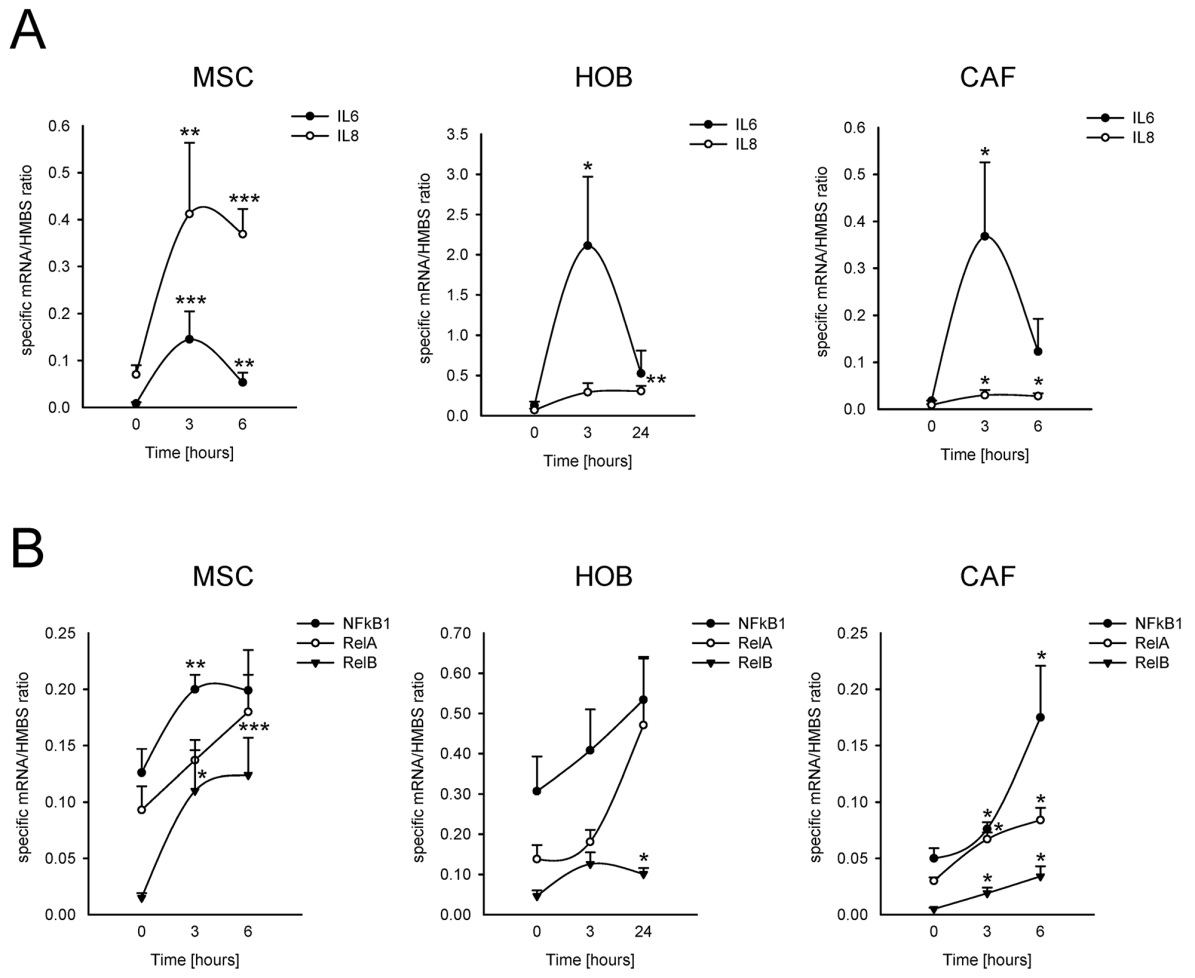
Short-term acidosis activates the expression of inflammatory mediators in BM-associated mesenchymal cells Graphical representation of mRNA expression over the time, starting from the acidosis exposure (medium at pH 6.8), of IL6 and IL8 **(A)** and of NF-κB transcription factors **(B)** in normal stromal cells, as revealed by Q-RT-PCR analysis. Mean ± SE (for MSC and HOB, N = 3 different lots, and for each lot, N =2 biological replicates, and N = 2 technical replicates; for CAF N = 1 lot, N = 2 biological replicates, N = 2 technical replicates). ***p<0.001, **p<0.01, and *p<0.05 versus the expression of the specified gene at the timing immediately before medium change with acidic medium in the same cells.

The incubation of MSCs at a low pH for 24 h induced the mRNA transcription of nociceptive mediators (NGF and BDNF) and inflammatory cytokines (CCL20, CCL3, CCL5, IL15, IL1a, IL1b, IL23A, IL34, IL6, IL8, TNF, TGFA; Figure 4). Q-RT-PCR in MSCs, HOBs, and CAFs partially confirmed the deep-sequencing results (Figure 5A); the low pH caused a significant increase in BDNF (MSCs *p*=0.025, HOBs *p*=0.0039, CAFs *p*=0.0209), NGF (HOBs *p*=0.0039, CAFs *p*=0.0209), CCL5 (MSCs *p*=0.0003, CAFs *p*=0.0209), IL1b (MSCs *p*=0.0039), IL6 (MSCs *p*=0.0002, HOB *p*=0.0209, CAFs *p*=0.0202), and IL8 (MSCs *p*=0.0018, CAFs *p*=0.0356). The expression levels of the nociceptive mediators NGF and BDNF were higher than those in the tumor cell line bmMDA under acidosis (Figure 5B), and of cells of neuronal origin at pH 7.4 (Figure 5A). Protein analysis confirmed the secretion of IL6 and IL8 into the media (IL6 *p*=0.0177 for MSCs, *p*=0.0039 for HOBs, and *p*=0.0209 for CAFs; IL8: *p*=0.0116 for MSCs, *p*=0.0039 for HOBs, and *p*=0.0209 for CAFs; Figure 5C). Furthermore, the induction of IL6 and IL8 mRNA expression by the low pH was very rapid, appearing after only 3 h in each cell type (Figure 6A), and was possibly mediated by NF-κB pathway activation because either NF-κB1, RelA, or RelB mRNA significantly increased at similar time-points, especially in the MSCs (Figure 6B). In contrast to untransformed cells, the breast cancer cells had a very low expression of the cytokines, at both at neutral (IL6: 0.004±0.002; IL8: 0.044±0.005) and acidic (IL6: 0.004±0.001; IL8: 0.049±0.007) pH values.

**Figure 7:**
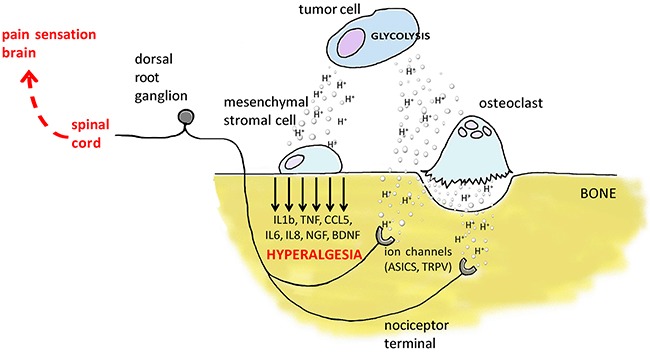
Graphical summary of the proposed model of the induction of pain and of hyperalgesia and nociceptor sensitization in the BM microenvironment In the BM microenvironment, carcinoma cells and tumor-induced osteoclasts pump in the extracellular space a high amount of protons that on one side stimulate ion channels on the membrane of the nociceptor terminal in bone, on the other side, promote the release of inflammatory and nociceptive mediators from the tumor-associated mesenchymal cells, including MSCs, HOBs, and CAFs that further enhance CIBP by promoting hyperalgesia and nociceptor sensitization.

**Figure 8:**
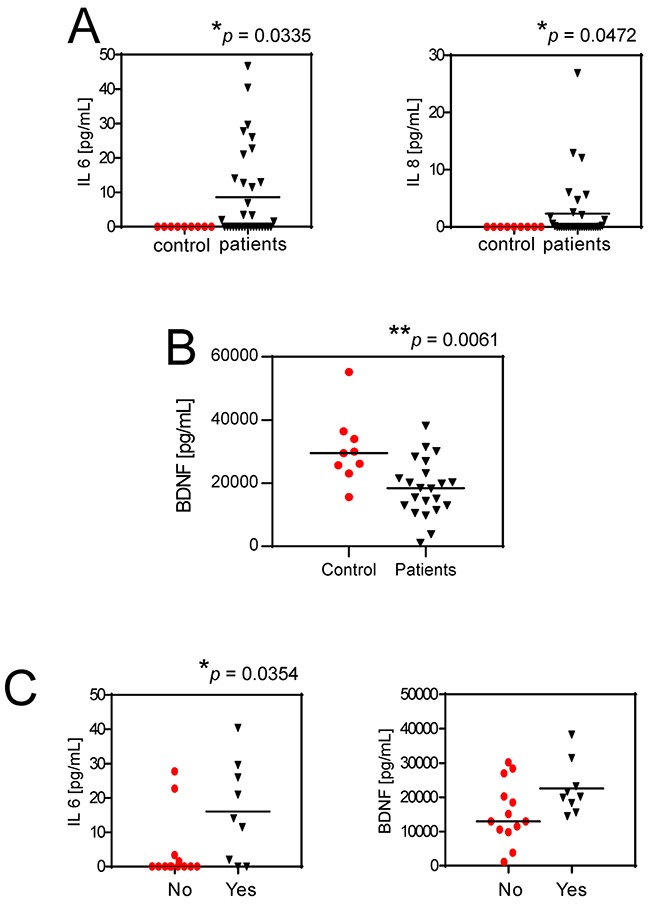
Serum levels of IL6 and IL8 and pain **(A)** IL6 and IL8 serum levels in BM patients versus healthy control subjects. Mean ± SE (N = 36 for patients, and N = 9 for controls, *p<0.05); **(B)** Serum levels of BDNF in patients with BM in respect to healthy control donors. Mean±SE (n=22 for patients, and N = 9 for controls, **p<0.01); **(C)** IL6 and BDNF serum levels in respect to breakthrough pain in BM patients (yes, patients suffering for breakthrough pain; no, patients not suffering for breakthrough pain). Mean ± SE (N = 13 and N = 9, respectively, *p<0.05).

Our data suggest a novel mechanistic model for untreatable CIBP that involves mesenchymal cells in patients with BM: the acidification of the BM microenvironment activates an auto-feeding process of pain by inducing of the secretion of nociceptive and inflammatory modulators from mesenchymal cells and thereby promoting hyperalgesia, leading ultimately to continuous sensitization of the nociceptors in the bone (Figure 7). The model matched evidence obtained from clinical samples. The serum levels of IL6 and IL8 in patients with BM of breast, lung and undefined carcinoma were significantly higher than those in healthy controls (*p*=0.0335 and *p*=0.0472, respectively; Figure 8A). Furthermore, although BDNF serum levels were lower in patients versus control (Figure 8B), high serum levels of IL6 and BDNF as well as high IL6 level alone were significantly correlated in patients with breakthrough pain vs patients without breakthrough pain (*p*=0.0131 and *p*=0.0354, respectively; Figure 8C). High serum levels of IL6 and IL8 in patients with cancer are assumed to be derived from strong local secretion from tumors. In contrast to that assumption, *in vitro* analysis and deep-sequencing screens including breast cancers (MCF7, T47D, and SKBR3 cell lines, data not shown), revealed consistent and strong expression of IL6 and IL8 by mesenchymal stromal cells and not by carcinoma cells, particularly when the mesenchymal cells were exposed to extracellular acidosis.

**Figure 9:**
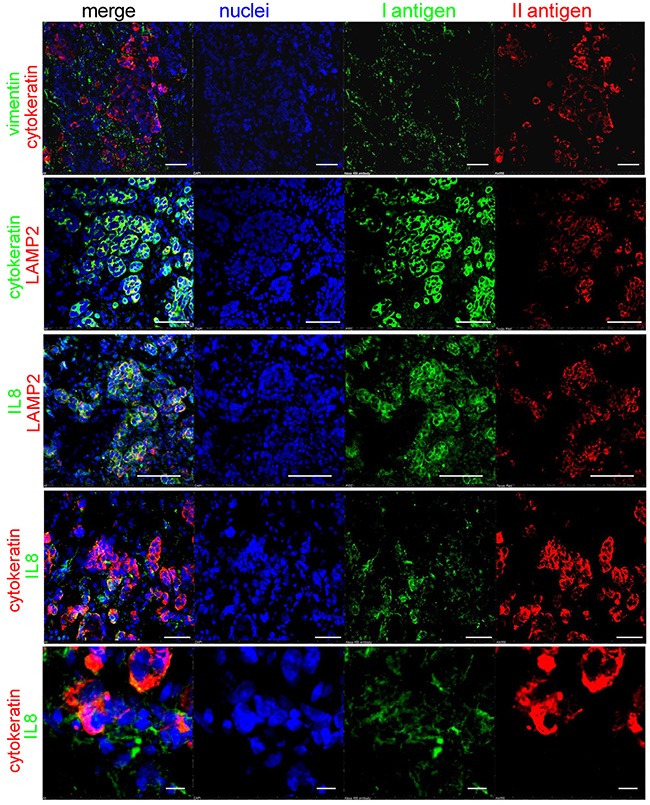
IL8 localization in BM tissue samples Immunofluorescence staining with different antigens and confocal analysis of a BM sections from a patient with a previous diagnosis of breast cancer. Intratumoral acidosis was revealed by LAMP2 staining. Cells were counterstained with Hoechst 33258 for nuclear blue signal (white bar corresponds to 50 μm, but in the last string at the bottom that corresponds to 10 μm).

We looked for IL8 localization in tissue sections of BM from breast carcinoma and also in relation to intratumoral acidosis. We used cytokeratin and vimentin staining to identify the carcinoma cells and the surrounding mesenchymal stroma, respectively (Figure 9, first string from the top). We used LAMP2 staining as a marker of the acidic microenvironment [19], which was confined to the carcinoma cells, as indicated by a complete overlapping with the cytokeratin signal (Figure 9, yellow color in the merged image of the second string). Similarly, IL8 localized very closely to the LAMP2-positive tumor area, but with only a partial overlap (Figure 9, third string), possibly because IL8 is secreted close to the acidic area of the BM microenvironment and mostly by untransformed mesenchymal stromal cells. The IL8 and cytokeratin staining only a partially overlapping (Figure 9, fourth string), as was more clearly evident at a higher magnification (Figure 9, fifth string).

## DISCUSSION

The identification of novel therapies that specifically and better target the pathogenic mechanism of CIBP more effectively than the currently available therapies is an urgent challenge. Within that context, the acidification of the interstitial pH of BM microenvironment into acidic values might play a major role because sensor neurons in the bone express acid-sensing nociceptors [20].

Recent studies suggest that CIBP is directly caused by bone resorption due to proton secretion by bone-resorbing osteoclasts, the altered metabolism of highly glycolytic and acidifying tumor cells, and the tumor-promoted inflammation that further enhances tissue acidosis [20, 21]. We analyzed and confirmed the acidification activity of carcinoma cells, with particular regard for those that form BM. As a preclinical model, we used a subclone of the triple-negative breast carcinoma cell line MDA-MB-231, which has a higher tendency than the parental cells to form osteolytic lesions in xenografts [16]. The bmMDA cells showed a greater ability and aptitude than the parental cells to secrete protons and to express ion/proton transporters related to the acid-base balance (CA9, MCT4, and V-ATPase V_1_G_1_). The increased acidification activity derives from a high rate of glycolysis, as indirectly suggested by higher GLUT-1 expression and lactate release. The glycolytic activity and the extracellular acidification potential of both bmMDA cells and MDA cells were further enhanced by hypoxia which was not unexpected because low oxygen tension further promotes glycolysis. The bone marrow is a quite hypoxic tissue (1-6% O_2_) [22], and hypoxia is a crucial microenvironmental factor that provides a platform for circulating breast carcinoma cells to colonize the bone and form BM [23]. Our data are in agreement with previous studies showing that the tendency of breast carcinoma to invade and form distant metastases strongly depends on tumor acidification [15], and on highly acidic lysosomes [24]. The acidification of extracellular space is a selective advantage in bone because it enhances the activity of extracellular-matrix degradation by cancer-secreted metalloproteinases [12, 25], and is associated with increased invadopodia formation [26] and with the induction of osteoclast-mediated bone resorption [27, 28], ultimately leading to an enhancement of erosion of the bone matrix [12]. Both tumor cells and osteoclasts acidify the extracellular space using V-ATPase, which is expressed on the lysosomal and plasma membranes [14, 24]. V-ATPase expression, and particularly that of the V_1_G_1_ subunit, which is highly expressed in bmMDA cells, has been related to tumor invasiveness [12, 24] and worse prognosis [29].

Previous attempts to target CIBP with bafilomycin A1, a toxic V-ATPase inhibitor, were successful in preclinical settings [30]. Therefore, we planned to target tumor extracellular acidification and acid-induced pain with omeprazole. Omeprazole and related drugs inhibit the grastric H^+^-ATPase and can also target V-ATPase at high concentration, through the binding to the V1A subunit [13, 31, 32], and are widely prescribed as pivotal treatments in association with NSAIDs to reduce gastric side effects in advanced cancer patients. Once we confirmed *in vitro* that omeprazole significantly reduced lysosomal acidification of both osteoclasts and bmMDA cells, we treated BM and corresponding CIBP [33] with omeprazole intraperitoneally and at a higher dose than the standard dose conventionally used in humans. Omeprazole reduced pain 2 weeks before the inhibition of osteolysis with no evident side effects. It comes as no surprise that osteolysis was affected later than pain as the pathogenic mechanism of osteolysis in BM is a more long-lasting process than the neuronal stimulation. Our results on preclinical models suggest that the use of anti-acid or anti-glycolytic therapies may be effective for the future to treat CIBP in patients with bone metastasis. In this context, the use of i.g. proton pump inhibitors, as we explored, or of rapamycin, an already clinically available drug that has been demonstrated to decrease lactate production and acidosis by decreasing glycolysis [34, 35], is very promising.

CIBP is a very complex phenomenon that is poorly understood. CIBP is the result of background pain combined with breakthrough pain that is rapid in onset and transient in nature. In contrast to background pain, breakthrough pain cannot be controlled by conventional analgesics [36] and, hence, interferes greatly with aspects of life [6]. Breakthrough pain has been more associated with CIBP than background pain. We speculated that, especially for the breakthrough component, pain stimulation and sensitization are related to the effect of acidity on the different cellular elements of the BM microenvironment. Indeed, in addition to being important algogenic mediators [7], protons might induce an inflammatory response in the tumor-associated reactive mesenchymal stroma, leading to the release of a plethora of inflammatory modulators at the injured site. Tissue acidification is commonly associated with inflammation because it acts as a driving force for mesenchymal stem cells during the regenerative process [37]. Similarly, in cancer, cells of mesenchymal origin are important components of the general host response to tumor-induced tissue damage, including tissue acidosis [38]. Osteoclasts [7, 39, 40] and cells of mesenchymal origin share with cells of neuronal origin the expression of acid-sensors. For example, osteoblasts express ASICs [39] and TRPV1 [7]. Thus, mesenchymal cells can perceive a low extracellular pH like nociceptors can. We also confirmed the expression of ASIC3 and ASIC4 acid-sensing channels, as well as GPRs in all the mesenchymal cells analyzed, MSCs and osteoblasts, and CAFs that were directly isolated from BM of breast carcinoma. Furthermore, in osteoblasts, extracellular acidosis enhanced the expression of GPR65 and ASIC4. To perform this type of *in vitro* assays, we used pH 6.8 since, although osteoclast activity might produce a pH around 3, this very high concentration of protons is compartmentalized in the Howship lacunae, and released in the extracellular space only at the end of the resorption activity, and immediately buffered with less acidic extracellular fluids.

Once mesenchymal cells detect extracellular acidosis, in addition to the release of metalloproteinases [41] that can concur to bone degradation, it may follow the activation and subsequent release of inflammatory mediators that might impact both on the tumor-related immune response and on pain induction (Figure 7). Indeed, we observed a consistent increase of CCL5, IL1b, IL6, and IL8. That induction was partially mediated by NF-kB pathway activation, as NF-κB nuclear translocation was increased. The secretion of cytokines and chemokines has been clearly associated with the modulation of nociceptor activation and sensitization that, in turn, are responsible for the transmission of inflammatory pain and for evoking even greater responses [42, 43]. For a patient that means that a painful stimulus (allodynia) generates greater pain (hyperalgesia). Furthermore, recent studies have pointed out that cells of mesenchymal origin can directly release neuromodulators. For example, during bone damage, cartilage cells release nerve growth factor (NGF) [44]. Similarly, MSCs can release the neurotrophic factors NGF and BDNF [45] and, according to our data, their expression is potently induced by extracellular acidosis. NGF and BDNF regulate the survival, development, and function of sensory and sympathetic neurons, which are essential for the retention and generation of pain [43]. NGF evokes inflammatory hyperalgesia by promoting changes in the conductive properties of axons, by modulating the direct phosphorylation and membrane trafficking of TRPV1 by TrkA, and by modulating the expression of Na channels and ASIC [46]. Hence, the increased expression of ASIC4 that we observed in acid-induced MSCs might derive from an NGF autocrine pathway. Finally, it was clearly demonstrated in a rat model of CIBP that BDNF induces and maintains hypersensitivity [47]. In conclusion, according to our results, it is possible to assume that in patients with BM and suffering for pain, CIBP is also mediated by extracellular environment that in bone, in respect to the primary site, is particularly more acid due to the presence of both active tumor cells and osteoclasts, and is possibly differently innervated with acid-sensitive nerve fibers. As a consequence of the alterations of the tumor microenvironment, the acid-induced release of inflammatory mediators and neuromediators also possibly explains the lack of responsiveness to conventional anti-analgesic drugs in patients with BM and with breakthrough pain. Hence, in addition to anti-acid strategies, the use of anti-NGF antibodies that are in the clinical phase of development for pain relief could be explored [48]. In agreement with our hypothesis, human studies indicated that the tumor microenvironment causes an abnormal remodeling of nearby sensory fibers, which is perceived as pain by patients [43]. Furthermore, previous literature reported high circulating levels of IL6 and IL8 in patients with BM [49], and that patients suffering from pain with advanced breast cancer have high systemic levels of the inflammatory mediators TNF, IL-1b, and IL6 [50]. Similarly, serum concentration of IL6 and IL8 in patients with bone metastasis of breast, lung and undefined carcinoma that we enrolled had significantly higher levels for IL6 and IL8 than healthy controls.

To specifically assess the correlation between biomarkers in clinical samples and the biomarkers that we identified in mesenchymal cells *in vitro*, we quantified the serum levels of BDNF; however, those showed an opposite trend possibly because low levels of BDNF have been associated with depression and anxiety, which are quite common in patients with BM [51, 52]. Despite that, we found a trend of increased BDNF and a significant increase of IL8 in patients with breakthrough pain. Furthermore, when we considered IL6 together with BDNF, the combined high serum levels of those two biomarkers were significantly correlated with breakthrough pain.

Finally, the analysis of tissue sections of BM from breast carcinoma suggest that carcinoma cells are highly acidic, as demonstrated by the strong LAMP2 staining [19] in carcinoma cells, and furthermore, that IL8 appeared to be secreted for the most part by the mesenchymal stroma, close to the acidic area of the tumor, rather than by the tumor itself. Therefore, the higher levels of IL8 in patients with advanced cancer with BM and breakthrough pain possibly and partially originate from the mesenchymal stroma adjacent to the tumor cells and within the acidic microenvironment.

In conclusion, we have pointed out for the first time that intratumoral acidosis and the mesenchymal stromal reaction to tumor-associated acidosis might trigger CIBP, especially in BM, underlining once more that cancer-associated fibroblasts and mesenchymal stromal cells coming from the host are of the foremost importance in cancer progression. With this study, we offer intratumoral acidosis as a promising target for novel and more effective palliative treatments for patients with advanced cancer.

## MATERIALS AND METHODS

### Reagents

Unless otherwise indicated, all reagents were obtained from Sigma: IMDM, penicillin, streptomycin, Trizol, unbuffered RPMI-1640 (Life Technologies); anti-fibroblast magnetic microbeads (Miltenyi Biotech); FBS, DMEM (Euroclone); glucose (Merk); MuLV Reverse Transcriptase (Applied Biosystems); RANKL, M-CSF (Peprotech); nitrocellulose membrane, D-luciferin (Thermo Fisher Scientific); BCA protein assay, Restore Western Blot Stripping buffer, Pierce ECL 2 Western Blotting Substrate (Pierce); TruSeq RNA Sample Prep Kit v2, Cycle Sequencing v4 regents (Illumina); Human IL6 DuoSet ELISA Kit, Human CXCL8/IL8 Quantikine ELISA Kit, Human Free BDNF Quantikine ELISA kit (R&D Systems Inc.). The antibodies used were: anti-rabbit Alexafluor, anti-mouse Alexafluor (Molecular Probes); Anti-MCT4 (sc-50329), anti-TBP (sc-204, Santa Cruz Biotechnology); anti-cytokeratin (M0821, Dako); anti-cytokeratin-TRITC (41-9003, Affimetrix eBioscience); anti-LAMP2 (HPA029100); anti-IL8 (AB18672, Abcam); anti-vimentin (sc-6260, Santa Cruz Biotechnology); omeprazole (Sandoz); ketamine (MSD Animal Health Srl). Horseradish peroxidase-conjugated anti-mouse and anti-rabbit were obtained from GE Healthcare.

### Cell cultures

SH-SY5Y and MDA-MB-231 (MDA) cell lines were purchased from the American Type Cell Culture Collection (ATCC). The subclone (bmMDA) was previously derived from the parental MDA cell line [16, 53]. We used mesenchymal stromal cells (MSCs) from bone marrow (BM-MSCs, 5 lots, Lonza) and from adipose tissue (AD-MSCs, 1 lot, ATCC), osteoblasts (HOBs, 1 lot, VWR International PBI) from healthy donors, and cancer-associated fibroblasts (CAFs) as models of the human mesenchymal stroma of BM.

Human skin fibroblasts (Fb, 2 lots) were provided by the Japanese Collection of Research Bioresources. We isolated CAFs from BM biopsies using anti-fibroblast magnetic microbeads after obtaining signed informed consent and approval from the Rizzoli Orthopaedic Institute Ethical Committee (n. 0037602 of 14.11.2013). To obtain CAFs, we mechanically minced BM samples of breast carcinoma, and the obtained cell suspension was then seeded. When semi-confluence was reached, after cell trypsinisation, we used anti-fibroblast magnetic microbeads and MiniMACS™ separation (Miltenyi Biotec). CAF phenotype was confirmed by the analysis of smooth muscle alpha (α)-2 actin (ACTA2) and Fibroblast Activation Protein Alpha (FAP) expression in respect to normal human skin fibroblasts, at mRNA level (Supplementary Figure 2, *p=0.0209 for both ACTA2 and FAP), with Q-RT-PCR. Osteoclast cultures were derived from buffy coats of healthy donors (AVIS, Bologna) [54, 55]. To obtain osteoclast **c**ultures, buffy coats were collected after their expiration date (1 days after harvesting). Peripheral blood mononuclear cells (PBMC) from healthy donors were layered on Ficoll-Hystopaque gradient. The isolated mononuclear precursors were then seeded in 8-well chamber slides (3×10^6^ cells/cm^2^) in DMEM supplemented with glucose [25 mM], 10% characterized FBS, plus penicillin [20 U/mL], streptomycin [100 mg/mL]. After 1 h, non-adherent cells were removed and replaced with new medium added with RANKL [50 ng/mL] and M-CSF [10 ng/mL] (differentiation medium). In order to verify osteoclast differentiation, after 5 to 7 days of cell culture, cells were analyzed for tartrate-resistant acid phosphatase (TRAP) activity by Acid Phosphatase Leukocyte assay and nuclei were stained with Hoechst 33258. Only TRAP positive cells with more than 3 nuclei were considered as osteoclasts.

MDA, bmMDA, and SH-SY5Y were maintained in complete IMDM, MSCs in complete Alpha-MEM, HOBs and CAFs in complete DMEM, and osteoclasts in complete DMEM high glucose medium. Cells were cultured at 37°C, 5% CO_2_. Complete media were supplemented with 10% FBS, plus 100 units/mL penicillin, and 100 mg/mL streptomycin. Unless otherwise indicated, primary cultures and cell lines were maintained at pH 7.4. To create acidic conditions, we adjusted the pH of the medium to 6.5 or 6.8 using specific concentrations of sodium bicarbonate as previously described [18]. At the end-point of each experiment, we measured the pH of the culture supernatants to confirm the maintenance of the prefixed pH values during the incubation period.

We used only MSCs, HOBs, and CAFs that had been passaged no more than six times. Osteoclasts were used only at passage 0.

### Q-RT-PCR

Total RNA was extracted using Trizol and reverse transcribed using MuLV Reverse Transcriptase. Specific genes were amplified by Real-Time PCR. The primers and probes are listed in Table 1. For RNA isolation under hypoxia, we incubated the cells with 1% O_2_ (*In vivo* 2-400 hypoxic incubator; Ruskin Technologies) for 24 h. Real-Time PCR was performed by amplifying 1 μg of cDNA using the Light Cycler instrument and the Universal Probe Library system (Roche Applied Science). Probes and primers were selected using the web-based assay design software (ProbeFinder: http://www.roche-applied-science.com). Results were normalized to Hydroxymethylbilane synthase (HMBS) or beta-Actin (β-actin), according to the 2^−ΔΔCT^ method. We performed each assay with at least two technical replicates and two biological replicates.

**Table 1:** Probes and primers

Gene	Full name	Accession Number	Primers	Probe
**HMBS**	Hydroxymethylbilane synthase	NM_000190.3	F = tgtggtgggaaccagctcR = tgttgaggtttccccgaat	26
**CA9**	Carbonic anhydrase 9	NM_001216.2	F = tgcctatgagcagttgctgtR = ccagtcctgggacctgagt	73
**GLUT1**	Glucose transporter-like protein I	AY034633.1	F = ggttgtgccatactcatgaccR = cagataggacatccagggtagc	67
**MCT4**	Solute carrier family 16 member 4	NM_001206952.1, NM_004207.3, NM_001042422.2, NM_001042423.2, NM_001206950.1, NM_001206951.1	R = gagtttgggatcggctacagF = cggttcacgcacacactg	58
**BDNF**	Brain-derived neurotrophic factor	NM_170731.3, NM_170732.3, NM_170733.2, NM_170734.2, NM_170735.4, NM_001709.3	R = gtaacggcggcagacaaaF = gaccttttcaaggactgtgacc	86
**NGF**	Nerve growth factor	NM_002506.2	R = agtggtcgtgcagtccaagF = ggacattacgctatgcacctc	42
**CCL5**	Chemokine (C-C motif) ligand 5	NM_002985.2	F = tgcccacatcaaggagtatttR = ctttcgggtgacaaagacg	59
**IL1B**	Interleukin 1, beta	NM_000576.2	R = aaagcttggtgatgtctggtcF = ggacatggagaacaccacttg	10
**TNF**	Tumor necrosis factor	NM_000594.2	R = cagcctcttctccttcctgatF = gccagagggctgattagaga	29
**IL6**	Interleukin 6	NM_000600.3	F = gatgagtacaaaagtcctgatccaR = ctgcagccactggttctgt	40
**IL8/CXCL8**	C-X-C motif chemokine ligand 8	NM_000584.3	F = gagcactccataaggcacaaaR = atggttccttccggtggt	72
**ACCN4**	Acid sensing ion channel subunit family member 4	NM_182847.2	R = gacagatgggcctgttcattF = ccatacccgcttcagtcg	67
**ACCN3**	Acid sensing ion channel subunit 3	NM_004769.2	R = ccttgtgggcctgagaacF = cccatgccacccctagta	43
**GPR65**	G protein-coupled receptor 65	NM_003608.3	R = gggcagatcgccttaggtF = caggtctggcacatttttga	54
**GPR4**	G protein-coupled receptor 4	EU432116.1	R = ttccgccatccctctacatF = ccacagagccaggcagtt	44
**ATP6V1B2**	ATPase H+ transporting V1 subunit B2	NM_001693.3	R = tggccgaagacttccttgF = ccgaaatgccagtctgaatc	6
**ATP6V0C**	ATPase, H+ transporting, lysosomal 16kDa, V0 subunit c	NM_001694.2	R = ttcgtttttcgccgtcatF = ccactgggatgatggacttc	76
**ATP6V1G1**	ATPase H+ transporting V1 subunit G1	NM_004888.3	R = tcagtctcaggggattcagcF = tcagcctgagcttcttctttg	75
**ACTA2**	Actin, alpha 2, smooth muscle, aorta	NM_001141945.1, NM_001613.2	R = ctgttccagccatccttcatF = tcatgatgctgttgtaggtggt	58
**FAP**	Fibroblast activation protein, alpha	NM_004460.2	R = tggcgatgaacaatatcctagaF = atccgaacaacgggattctt	19
**NFKB1**	Nuclear factor kappa B subunit 1	NM_003998.3	F = cctggaaccacgcctctaR = ggctcatatggtttcccattta	49
**RelA**	RELA proto-oncogene, NF-kB subunit	NM_021975.3	F = actgtgtgacaaggtgcagaaR = cacttgtcggtgcacatca	64
**RelB**	RELB proto-oncogene, NF-kB subunit	NM_006509.3	F = gattgtcgagcccgtgacR = ccacgccgtagctgtcat	4

### Western blotting

We lysed the cells at semi-confluence with boiling buffer containing 1% sodium dodecyl sulfate (SDS), Tris pH 7.4 (20 mM), 2-Mercaptoethanol (5%), and sodium orthovanadate (1 mM). We then quantified the protein content by BCA assay. Equal amounts of proteins were subjected to SDS-PAGE, blotted on nitrocellulose membranes, and then incubated with specific primary and secondary antibodies. The reactions were revealed by Pierce ECL 2 Western Blotting Substrate. To detect different proteins on the same blot, we stripped the nitrocellulose membranes with Restore Western Blot Stripping buffer and then reprobed them. We quantified the signals using the VisionWorksLS Analysis Software (Biospectrum, UVP). The results presented are representative of four biological replicates.

### Lactate assay

For the lactate assay, we plated 6×10^4^ cells/cm^2^ on 12-well plates in complete IMDM. After 24 h, we changed the media with new media containing 2% FBS. After 48 h, we harvested the culture supernatants and measured the L-lactate concentration using the EnzyChrom L-Lactate Assay Kit (BioAssay Systems, ECLC-100). For the hypoxic condition, we incubated the cells for 48 h after seeding as previously described. Normalization was performed with respect to the total protein content, as measured by BCA protein assay. The experiments were performed with four technical replicates.

### pHMed assay

We measured the pH of the culture medium using a micro-electrode [18]. Cells were (16×10^6^ cells) washed twice in pHMed solution (80% normal saline, 10% unbuffered RPMI-1640 and 10% FBS), and incubated in pHMed in suspension for 3 hrs at 37°C. Cells were then centrifuged (10 min at 500xg), and supernatant was collected for pH measurement. pH was immediately measured by a digital pH-meter (pH 301, HANNA Instruments). The difference of pH of pHMed solution alone (negative control) and the cell supernatants was analyzed. The experiment was repeated with six biological replicates.

### Confocal spectral analysis of the lysosomal pH

We used the emission spectra of the pH-sensitive dye acridine orange to measure slight pH variation in the intracellular acidic organelles [13]. Twenty-four h after seeding, cells were incubated at pH 6.5 for 30 min with or without omeprazole (100 μM) and then washed and analyzed. Osteoclasts at 7 days of culture or MDA or bmMDA at semi-confluence were incubated with acridine orange [0.5 μg/mL] for 15 min. After one wash, x,y emission spectra from a confocal section within a living cell were recorded using a confocal laser microspectrofluorimeter (Nikon, TI), equipped with an argon-ion laser. Cells were focused with x40 lens, 1.3 NA (S Fluor, Nikon) and excited at 457 nm, and the resulting fluorescence emission in the 500-700 nm range was collected. For intracellular measurements of acridine orange emission the pinhole size was fixed to a diameter of 54 μm. To characterize the profile of acridine orange emission spectra, the red band contribution (R%) within the whole emission spectrum has been calculated as follows: R%=100*I_655_/ (I_655_ + I_530_) where I_655_ and I_530_ are the green (520-540 nm) and the red (645-665 nm) integrated emission intensities, respectively. The R% was calculated for all the acidic organelles within a single cell, and the average R% of acidic organelles of one single cell was considered.

We averaged of the measured signals from all the lysosomes in at least fourteen single cells.

### *In vivo* study

Procedures involving animals and their care were conducted with institutional approval and conformed to national and international laws and policies (EEC Council Directive 86/609, OJ L 358, 1, Dec. 12, 1987; Italian Legislative Decree 116/92, Gazzetta Ufficiale della Repubblica Italiana n. 40, Feb. 18, 1992; NIH guide for the Care and Use of Laboratory Animals, NIH Publication No. 85–23, 1985). Four-week-old female BALB/c^nu/nu^ mice were anaesthetized with ketamine/xylazine (75 mg/Kg body weight + 15 mg/Kg body weight, respectively), injected monolaterally with bmMDA (5×10^4^ cells/10 μl PBS) in the medullar cavity of the left tibia and then treated i.p. with vehicle (0.9% NaCl) or omeprazole (40 mg/kg body weight). Treatment was administered 5 days per week for 4 weeks, starting the day after tumor cells inoculation.

To calculate the entity of osteolytic lesions we anaesthetised mice as described, laid them flat on a x-ray-sensitive film and exposed them in an x-ray cabinet (Faxitron, Tucson, AZ, USA) to obtain standard 2D x-ray scans. After developing the films, we took high resolution images of them and analysed osteolytic lesions by manually selecting the osteolytic areas using the software ImageJ (NIH).

For CIBP assessment, we tested the mice every week through the Linton instrumentation incapacitance tester (Supplementary Figure 3) [54]. Briefly, mice were placed in the measurement chamber in an upright position, with the non-inoculated and inoculated limbs placed on 2 separate micro-scales. 5 measurements of weight distribution between the two hindlimbs were carried out. Changes in hind paw weight distribution between the right (tumor bearing) and left (contralateral control) limbs were utilized as an index of joint discomfort due to tumor growth. The change in hind paw weight distribution was calculated by determining the difference (expressed as percent difference) in the amount of weight (g) between the left and right limbs.

### Illumina genome analyzer sequencing and data analysis

mRNA expression was evaluated by extracting total RNA using Trizol from cells cultured at pH 6.5 and ph 7.4 for 24 h. We quantified the RNA using a Bioanalyzer (Agilent). The RNA integrity numbers (RIN) and A260/A280 ratios were all equal to 10, and greater than 1.8, respectively. We converted the total RNA to a library of template molecules for high-throughput DNA sequencing using the TruSeq RNA Sample Prep Kit v2. We quantified the library using a Bioanalyzer. Library (7 pM) was subjected to cluster amplification to cluster generation on a Single Read Flow Cell v4 with a cluster generation instrument (Illumina). Sequencing was performed on a Genome Analyzer GAIIx for 76 cycles using Cycle Sequencing v4 regents. Image analysis and base calling were performed using Off-Line Basecaller Software 1.6 (Illumina). Reads were aligned using ELAND v2 of CASAVA Software 1.7 with the sequence data sets. Human genome build 19 (hg19) were downloaded from University of California, Santa Cruz genome browser (http://genome.ucsc.edu/) as the analytic reference. Transcript coverage for every gene locus was calculated from the total number passing filter reads that mapped, by ELAND-RNA, to exons. These analyses were performed using default parameters. The advanced analysis for quantification with Quantile normalization algorithm was performed using Avadis NGS software (version1.5, Strand Scientific Intelligence Inc., San Francisco, CA). The filtering was performed using default parameters.

We registered the obtained data at DDBJ/EMBL/GenBank (DRA004087 and DRA004091).

### ELISA assay

We quantified IL6, IL8, and BDNF in culture supernatants and serum samples using the Human IL6 DuoSet, Human CXCL8/IL8 and Free BDNF Quantikine ELISA Kits, respectively. To generate the supernatants, we seeded cells at 1.5×10^4^ cells/cm^2^, and after adhesion, incubated them for 20 h in Alpha-MEM plus 0.1% FBS. We then washed the cells and incubated them in acidic (pH 6.8) or physiological (pH 7.4) Alpha-MEM plus 0.1% FBS. After 24 h, the supernatants were collected. Normalization was performed with respect to the total protein content measured by BCA protein assay. We obtained blood samples from nine healthy controls and 36 patients with newly diagnosed BM at the time of diagnosis (7 breast carcinomas, 10 from lung carcinomas, n. 19 from undefined carcinoma) and that were seen at the Rizzoli Orthopaedic Institute or were enrolled from the ANT foundation in Bologna from February 2014 through July 2016. To identify patients suffering from breakthrough pain at presentation, we administered a pain questionnaire to a subgroup of 22 patients that suffering from pain (Appendix 1). Blood and pain questionnaires were collected at clinical presentation, before surgical treatment of the bone lesion, with signed informed consent and with institutional ethical committee approval (n. 0037602 of 14.11.2013). Breakthrough pain was considered short duration (less than 15 min) pain perceived in the last 24 h that significantly affected the quality of life [6].

### Immunofluorescence

We obtained tissue samples from 1 patient that was seen at the Rizzoli Orthopaedic Institute and with newly diagnosed BM at the time of diagnosis with a past diagnosis of breast carcinomas. Tumor tissue was collected during surgical treatment of the bone lesion, with signed informed consent and with institutional ethical committee approval (n. 0037602 of 14.11.2013). We embedded BM samples at optimal cutting temperature (OCT) and frozen them on dry ice before sectioning. We sectioned the samples at 10 μm and put them on a glass slide covered with 2% acetone in saline. The slides were stored at -80°C until immunostaining. Defrosted sections were permeabilized for 15 min with PBS containing 0.1% TritonX-100, blocked with 1% BSA for 1 h, and incubated with primary antibodies overnight. Then the sections were incubated with fluorochrome-conjugated secondary antibodies and Hoechst 33258 for nuclear staining and mounted and observed by confocal microscopy (Nikon TI-E).

### Statistical analysis

Because of the small number of observations, we did not consider the data to be normally distributed and therefore used non-parametric tests. We performed statistical analysis using the StatView 5.0.1 software (SAS Institute Inc.). For the difference between two groups, we used the Mann-Whitney U test. For the correlations between breakthrough pain and the simultaneous presence of high serum IL6 and high BDNF levels, we used the Fisher's exact test. We considered IL6 and BDNF levels high when they were above the 40th percentile of the patient group with pain (0 pg/mL and 22,519 pg/mL, respectively). For the *in vivo* studies Student's t-test was used with α=0.05. We expressed *v*alues as the means±SE and only *p*<0.05 values were considered to be statistically significant.

## SUPPLEMENTARY MATERIALS FIGURES



## Abbreviations

CIBP: Cancer-induced bone pain
NSAIDs: non-steroidal anti-inflammatory drugs
BM: bone metastases
ASICs: acid-sensing ion channels
TRPVs: transient receptor potential channel-vanilloid subfamily members
V-ATPase: vacuolar H+-ATPase
NF-kB: nuclear factor kappa-light-chain-enhancer of activated B cells
CA9: carbonic anhydrase 9
MCT4: monocarboxylate transporter 4
GLUT-1: glucose transporter 1
NGF: nerve growth factor
BDNF: brain-derived neurotrophic factor
MSCs: mesenchymal stromal cells
HOBs: osteoblasts
CAFs: cancer-associated fibro-blasts
Fb: fibroblasts
ACTA2: smooth muscle alpha (α)-2 actin
FAP: Fibroblast Activation Protein Alpha
PBMC: Peripheral blood mononuclear cells
TRAP: tartrate-resistant acid phosphatase
HMBS: Hydroxymethylbilane synthase
b-actin: beta-Actin
SDS: sodium dodecyl sulfate
OCT: optimal cutting temperature
