# Supplementary material for: Intratumoral acidosis fosters cancer-induced bone pain through the activation of the mesenchymal tumor-associated stroma in bone metastasis from breast carcinoma
Source: Oncotarget. 2017 Apr 13;8(33):54478–96. doi: 10.18632/oncotarget.17091 (PMC5589596; doi:10.18632/oncotarget.17091)
Supplement: Supplementary file 1 [file oncotarget-08-54478-s001.pdf]

Intratumoral acidosis fosters cancer-induced bone pain through the activation of the mesenchymal tumor-associated stroma in bone metastasis from breast carcinoma

SUPPLEMENTARY FIGURES AND APPENDIX

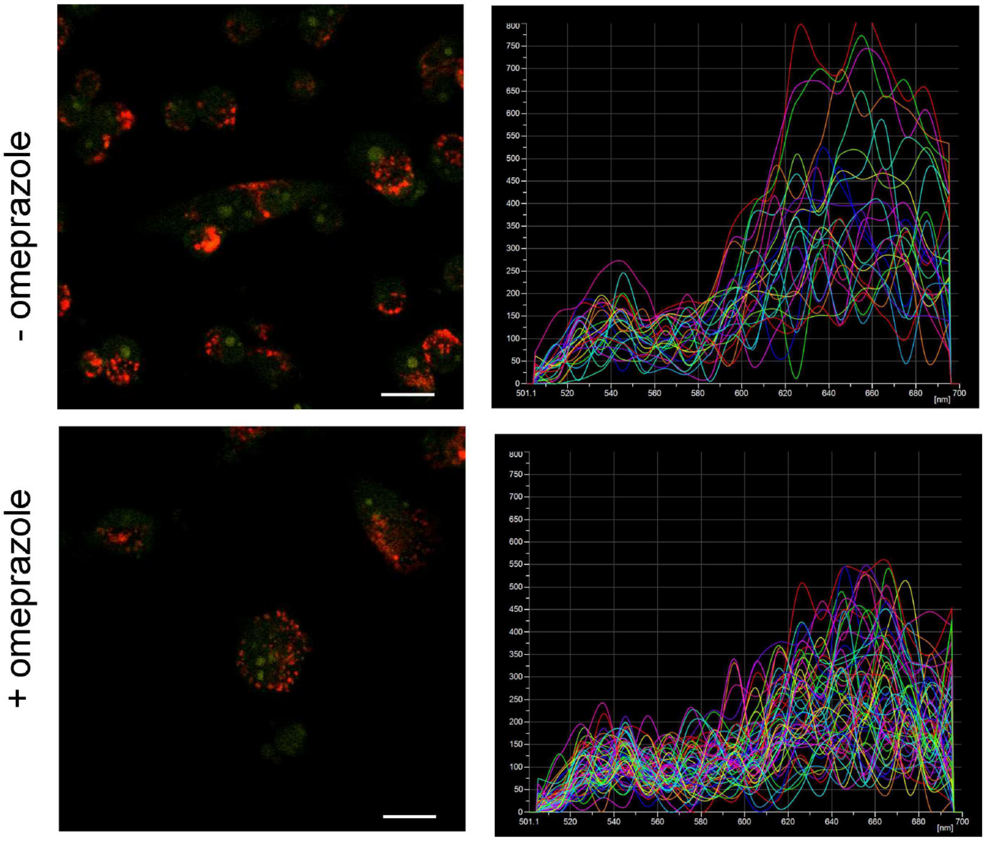

Supplementary Figure 1: Representative image of acridine orange staining (left panel) and graphical representation of the emission spectrum by confocal analysis (right panel) of all the lysosomes per cell of one representative cell of the bmMDA cell line, in the treated or not treated cultures with omeprazole.

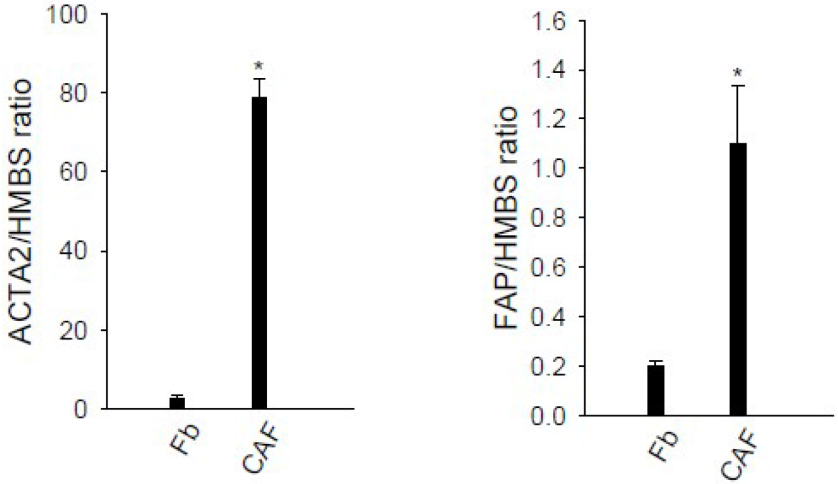

Supplementary Figure 2: Q-RT-PCR analysis of smooth muscle alpha (α)-2 actin (ACTA2) and Fibroblast Activation Protein Alpha (FAP) revealed that cancer-associated fibroblasts (CAF) isolated from a BM of human breast carcinoma have significantly higher expression of these markers than normal human fibroblasts (Fb). Mean ± SE (N = 2 biological replicates, N = 2 technical replicates). *$p < 0.05$.

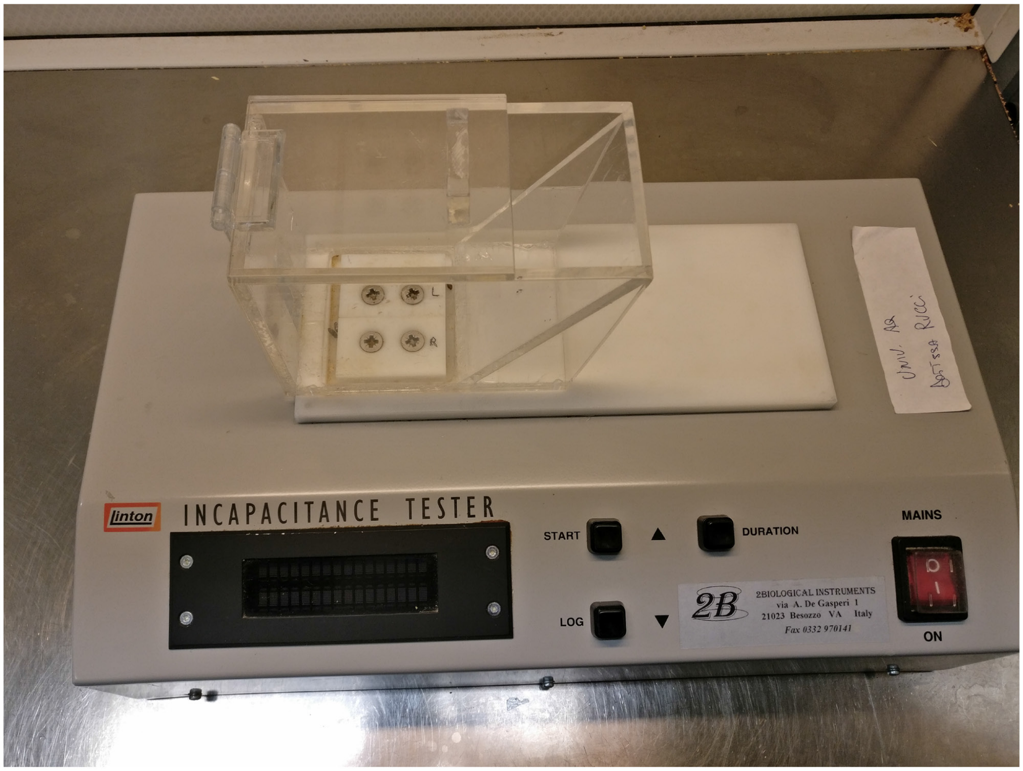

Supplementary Figure 3: The incapacitance tester used in this study, in order to assess the reduction of pain in xenograft orthotopic model of BM treated with omeprazole, both for both preventive and curative protocols.

Appendix 1:

1) In the last 24 hours, how severe was your worse pain? (please, circle how severe the pain has been).

0 1 2 3 4 5

2) In the last 24 hours, how severe was your weaker pain? (please, circle how severe the pain has been).

0 1 2 3 4 5

3) In the last 24 hours, on average, how severe was your pain? (please, circle how severe the pain has been).

0 1 2 3 4 5

4) In the last 24 hours, on average, how long does your pain last?:

(less than 5 min) (less than 15 min) (more than 15 min)

5) You're asked to rank on a scale of zero (non-functioning) to 10 (normal quality of life) how much your pain interfered during the last 24 hrs with:

your daily worklife: 0 1 2 3 4 5 6 7 8 9 10

your mood: 0 1 2 3 4 5 6 7 8 9 10

your ability to walk: 0 1 2 3 4 5 6 7 8 9 10

Your ability to work (inside and outside): 0 1 2 3 4 5 6 7 8 9 10

Your ability to socialize: 0 1 2 3 4 5 6 7 8 9 10

Your ability to sleep: 0 1 2 3 4 5 6 7 8 9 10

Your love of life: 0 1 2 3 4 5 6 7 8 9 10
